# An insight on promising strategies hoping to cure HIV-1 infection by targeting Rev protein—short review

**DOI:** 10.1007/s43440-021-00257-9

**Published:** 2021-04-11

**Authors:** Sahana Pai, Jayesh Mudgal, B. Venkatesh Kamath, K. Sreedhara Ranganath Pai

**Affiliations:** 1 Department of Pharmacology, Manipal College of Pharmaceutical Sciences, Manipal Academy of Higher Education, Manipal, Karnataka 576104 India; 2 Department of Pharmaceutical Biotechnology, Manipal College of Pharmaceutical Sciences, Manipal Academy of Higher Education, Manipal, Karnataka 576104 India

**Keywords:** Rev protein, HIV-1 infections, ABX464, Latent HIV-1 infection

## Abstract

Human immunodeficiency virus-1 (HIV-1) infection remains to be one of the major threats throughout the world. Many researchers are working in this area to find a cure for HIV-1. The group of the FDA approved drugs which are currently used against HIV-1 in the clinical practice include nucleoside reverse transcriptase inhibitors (NRTIs), non-nucleoside reverse transcriptase inhibitors (NNRTIs), integrase inhibitors (InIs), and protease inhibitors (PIs). Fixed dose combinations (FDCs) of these drugs are available and are used as per the anti-retroviral therapy (ART) guidelines. Despite these, unfortunately, there is no cure for HIV1 infection to date. The present review is focused upon describing the importance of a post-transcriptional regulatory protein “Rev”, responsible for latent HIV-1 infection as a possible, and promising therapeutic target against HIV-1.

## Introduction

The introduction of highly active antiretroviral therapy (HAART) benefited people infected with HIV-1 infections and allowed them to lead a normal lifestyle with increased life expectancy. Although HAART therapy successfully suppresses the viral replication to undetectable levels, there is an uncertainity of complete eradication of the virus from host. Furthermore, HAART also imposes the risk of adverse effects and unwelcomed drug interactions resulting in patient’s non-compliance to chronic HAART. Patients with ongoing HAART therapy may show undetectable viral load in clinical findings; however, it does not assure the cure from HIV-1. HIV-1 virus is capable of staying dormant in resting CD4+ cells and additional reservoirs (sanctuary sites) such as macrophages/ hematopoietic progenitor cells. This has been proved when latent HIV forms were identified in vivo in 1995. An interruption in the HAART therapy causes rebounds viremia, due to resumption of viral replication from its dormant form. These latent HIV forms usually emerge from virus reservoirs and sancturary sites. During HIV-1 replication, there are two latency phases, i.e., before (pre-integration latency) and after (post-integration latency) integration of viral DNA with host DNA [1, 2]. Host sanctuary sites, such as central nervous system, testes, macrophages, and CD4+ cells, play a vital role in failure of complete eradication of HIV-1 by antiretroviral (ARV) drugs [3, 4]. These sites serve as HIV reservoir, where ARV drugs fail to exert their optimal efficacy. Thus, it results in rebound viremia after stoppage of ARV therapy. This further may lead to persistent HIV-1 infections, which weakens the immunity of the patients. Eventually, the patient becomes more susceptible to multiple opportunistic infections and comorbid diseases which ultimately is fatal. Therefore, ongoing research is directed to find newer pharmacological targets against HIV-1. Recent findings claim to target HIV-1 virus latency by antisense inhibitors of the cellular miRNAs in HIV-1 reservoirs and sanctuary sites such as resting CD4 T+ lymphocytes by suppression of Tat and Rev protein [5]. The present review is an attempt to discuss the evidence, where efficacy of different molecules/drugs was tested against Rev protein to eradicate the latent HIV-1 infection.

## Methodology

Articles on HIV-1 proteins and their role in the HIV-1 life cycle were reviewed. More focused search was conducted on articles describing the Rev protein and its function, ABX464 therapy, persistent HIV-1 infections/rebound viremia, CRISPR/Cas9 technology, SiRNA directed HIV-1 inhibition, RNA aptamers, ribozymes, anti-Rev antibody, and Rev mutants. These articles were accessed using various databases, such as PubMed, Web of Science, and Scopus. The information regarding ongoing HIV-1 clinical trials on above-mentioned therapies was obtained from clinicaltrials.gov.

## HIV-1 proteins: a brief overview

HIV-1 encodes 15 distinct proteins which are categorised mainly into structural proteins (e.g., Gag and Env), accessory proteins (e.g., Nef, Vif, Vpr, and Vpu) and gene regulatory proteins (e.g., Tat and Rev) [6]. Apart from these, proteins which are encoded for viral specific enzymes are Pol for protease, RT for reverse transcriptase, and IN for integrase. Gag (assemblins), a structural protein that facilitates assembling and maturation of virion particles, has been reviewed to be the potential target [7]. However, Gag has not been successfully exploited to develop drugs. Nef, Vpu, and Env effectively downregulates the expression of CD4+ receptors present at the surface of helper T-cell [8]. Negative regulating factor (Nef), an accessory protein, is formed first inside HIV-1-infected cells. It is also primarily located in para-nuclear region and cytoplasm [8, 9]. Due to Nef, CD4 molecules undergo internalization followed by endosomal/lysosomal degradation. Nef mediates downregulation of MHC-1 resulting in suppression of antigen recognition, enhancing the replication of virus and therby reported to increases the virulence [10, 11]. Vpu is an auxiliary integral transmembrane protein produced at later stages of the infection [12, 13]. Vpu mediates the CD4 degradation by binding to the cytoplasmic domain of CD4 in the endoplasmic reticulum. This enables the assembling of virions by releasing Env precursor gp160 stuck with newly produced CD4 molecules in the endoplasmic reticulum. Early studies have also shown that Vpu enhances the virion release and the infectivity [14].

Tat is a small nuclear regulatory protein [6, 7]. Since 1985 after the Tat discovery [15], Tat has been found to play a key role in triggering transcription process of the viral genes for the pathogenesis and has been linked with viral latency [16]. It instigates the process of initiation and elongation (transcription) from long-terminal repeat (LTR) promoter region. Tat protein has shown various mechanisms of enhancing the HIV-1 infection. Some researchers have shown that Tat not only plays a role in LTR transcription, but also in regulating translation, thus affecting the cellular function [17]. It modulates the expression of various cytokines and chemokines which attracts the uninfected immune cells and macrophages to enhance the viremia via increasing viral entry and replication in these cells. In addition, several studies report that Tat can also contribute to the depletion of T cells during disease progression by up-regulating cellular pro-apoptotic gene [18].

Vpr is an accessory protein of 96 amino acids (14 kDa), introduced in the virion which plays a significant role in viral latent infection [19]. Vpr is considered to be a multifunctional protein. It allows the import of viral pre-integration complex (PIC) into the nucleus to facilitate the integration of viral genome with the host genome. In addition, it modulates T-cell apoptosis, transcriptional coactivation of viral and host genes and regulates nuclear factor kappa-B (NF-kB) activity to suppress the immune activation [20]. Vpr also upregulates HIV replication causing the G2 cell cycle arrest and promotes macrophage infection [21]. As Vpr promotes the viral infection by several functions, it becomes one of the promising targets for the researchers as a therapeutic intervention in HIV. Modulation of HIV-1 Rev expression by the biological and pharmacological inhibitors has been proposed as the effective strategy against the dormant form of HIV-1 in host reservoirs [5]. In the past decade, HIV-1 latency has attracted different approaches as a therapeutic cure of HIV-1 infection. Rev protein has emerged as a potential candidate against latent form of HIV-1. Therefore, there is need to understand the role of Rev and associated interventions against HIV-1.

## HIV-1 Rev protein

**Fig. 1:**
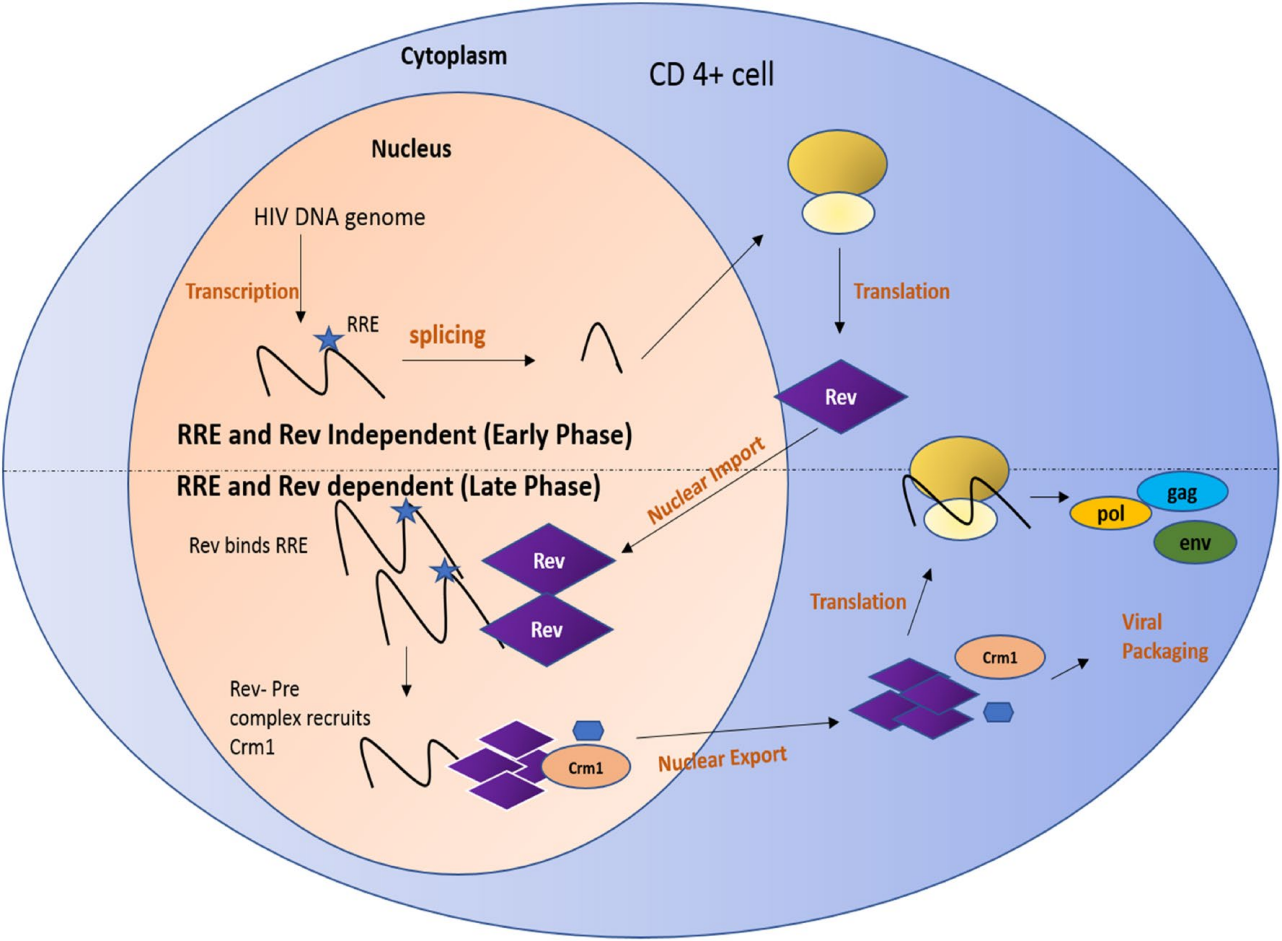
An overview of the role of Rev protein in the HIV life cycle [22, 23]

Rev protein is post-transcriptional regulatory protein which plays an important role in the HIV life cycle. Rev binds to Rev response element (RRE) and translocate un-spliced viral mRNA from nucleus to cytoplasm. This results in translation of structural proteins, such as Gag and Pol. For this process to occur, the concentration of Rev should essentially reach a threshold. In initial phases of HIV life cycle, the concentration of Rev is below threshold due to which the splicing signals are induced, and the viral mRNA gets spliced into 2 kb, 4 kb spliced mRNA which is not capable of producing structural proteins. In contrast, it can produce regulatory proteins, such as Rev and Tat. The Rev produced from the early phase is recycled back into the nucleus in the late phase which causes the Rev protein to reach the threshold (Fig. 1). Once the threshold is attained, Rev protein translocates the un-spliced viral mRNA from nucleus to cytoplasm which in combination with chromosome region maintenance (crm1) undergoes translation to produce structural protein gag, pol, and env. These proteins play a vital role in viral packaging and release of the matured virions [22, 23]. Latent HIV-1 infection can be prevented by various strategies which target Rev. These strategies are discussed below.

### ABX-464

**Fig. 2:**
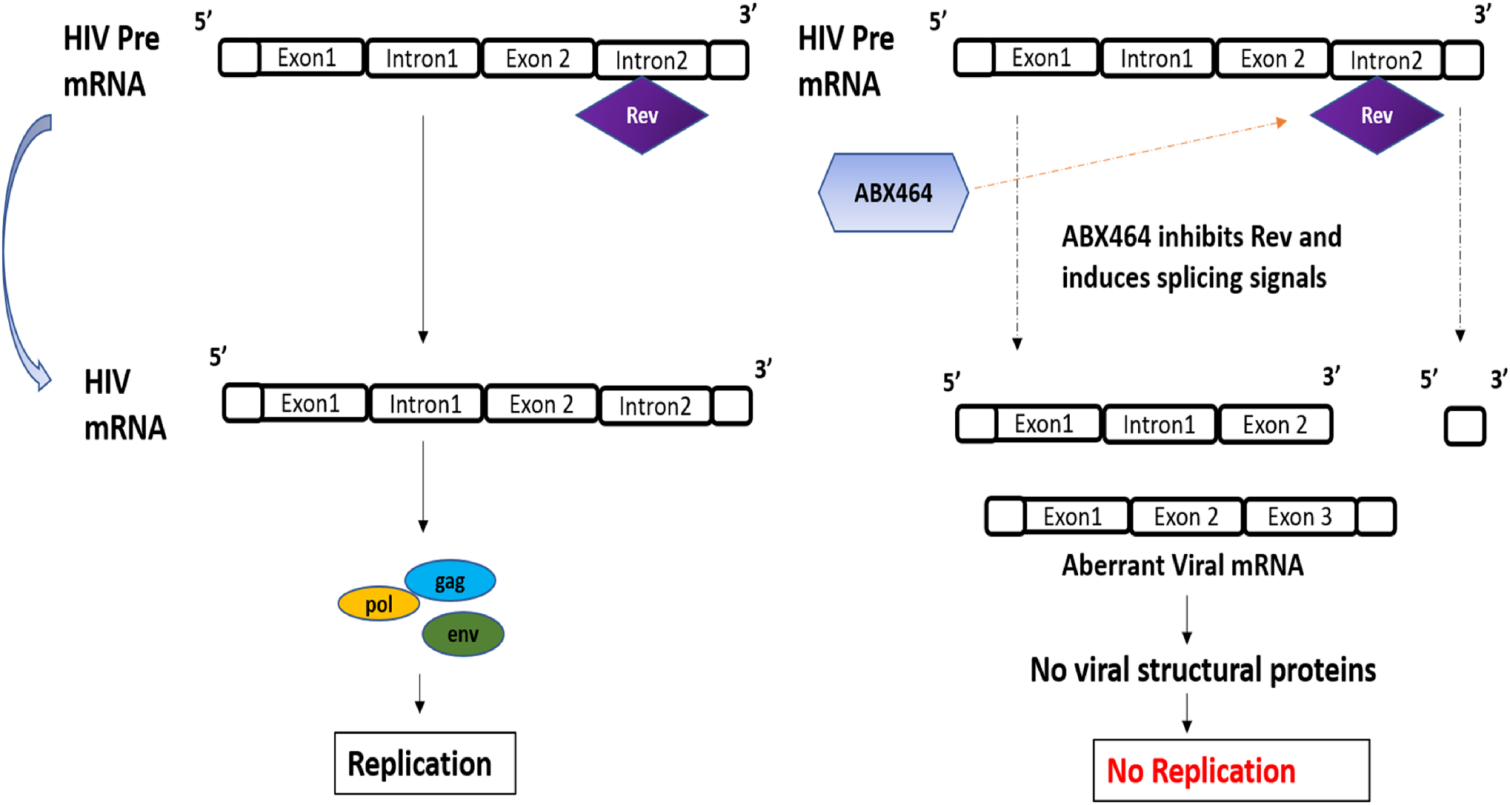
Mechanism of action of ABX464 [26]

As per the literature, ABX464 is one of the promising strategies in the treatment of HIV-1 infections. It has been found to be efficacious in preclinical and clinical trials [24–27]. This drug is currently in phase 2b clinical trial and the result published to date shows several advantages and few disadvantages. ABX64 targets viral RNA processes specifically and does not affect the human RNA processes. It does not allow HIV-1 virus mutation, which is the most common cause of rebound viremia thus preventing latent HIV-1 infections. It not only inhibits viral replication in CD4+ cells, but also reaches to in virus reservoirs, such as macrophages. Antiviral mechanism of action of ABX464 for regulating Rev expression has been depicted in the figure (Fig. 2). Furthermore, anti-inflammatory activity of ABX464 was published which may be beneficial in complications of HIV-1, such as inflammation. Literature also suggests that ABX464 upregulates miRNA-124 which plays an essential role in innate, adaptive immunity specifically it was proven to show cholinergic anti-inflammatory activity by dampening the pro-inflammatory cytokines, such as tumour necrosis factor (TNF)-α, interleukin-6, and MCP-1 production [25]. The metabolite of ABX464, i.e., ABX464-*N*-glucuronide, has a half-life of 160 h, and is also involved in inhibition of viral replication in macrophages [26]. However, a few disadvantages of ABX464 published to date include vomiting, nausea, mild epigastric pain, and pain in the abdomen. Further research and development is required to completely understand the concept of targeting regulatory proteins and results of clinical trials of ABX464 are awaited.

### CRISPR/Cas9

CRISPR/Cas9 tool is a widely used technology in genome engineering. Compared to many of the genome-editing techniques, Cas-9 (RNA-guided endonucleases) from the microbial adaptive immune system, clustered regularly interspaced short palindromic repeats (CRISPR) is prominently evolving. Utilizing the short RNA guide, CRISPR can be targeted against most of the genomic locations effectively. Applications of CRISPR/Cas9 technology have been widely explored in various therapeutic areas such as sickle-cell anemia, cystic fibrosis, Duchenne muscular dystrophy, cancer, hyperlipidemia, and also HIV-1 infection [27–29].

As per Lombardo et al., one of the ways to combat HIV infection is by inactivation of lymphocytic CCR5 receptor by non-homologous end joining (NHEJ)-mediated inactivation [30]. However, fresh approach in the utilization of CRISPR/Cas9 technology against HIV infection is reported with targeting the regulatory genes, such as Rev and Tat. In a study carried out by Opphini et al., the upcoming usage of this technique to specifically target HIV-1 regulatory genes and diminish the replication of viral particles has been described [31]. This study involved the construction and utilization of CRISPR/Cas9 via Lentiviral vector containing gRNAs with the potential to detect the exact sequences of DNA within the Tat and Rev coding sequences. Rev and Tat offer an excessive degree of viral gene expression in activated T cells during HIV-1 infection, and also preserve the provirus in latent state inside theresting T cells. Research also showed that inhibition of Rev and Tat protein and its role in stable Tat- and Rev-expressing 293 T cells. [31]. It was identified that the Cas9-associated mutation occurred within the Tat and Rev exon due to target site sequencing, while no mutation appeared in the related human genome sequences. This makes the therapy specific to inhibit the viral genome but not human genome.

In summary, CRISPR/Cas9 technology is effective in decreasing the replication of the HIV-1 proviral genome in latency models. The efficacy depends primarily on how far the gRNA sequence fits the target DNA and how advantageous to this effect is the targeting of the highly conserved regulatory genes tat and rev [32].The utilization of multiple gRNAs mixtures may also increase the effectiveness and reduce the chances of resistance. Even though many researchers provided evidences for the efficacy of CRISR/Cas9 in cell cultures, in vivo and ex vivo, its effectiveness and safety in humans are yet to be explored.

### Ribozymes

Ribozymes are the RNA molecules with potential enzymatic activity. They not only act as catalysts in specific biochemical reaction, but also has an essential role in RNA splicing and Viral Replication. There are various types of ribozymes especially hammerhead type and hairpin ribozymes [33]. Although the application of hammerhead-type ribozymes in the treatment of HIV1 infection was discovered in early 1990s, the utilization of the ribozymes in this therapeutic area is still being explored. Extensive research has been carried out in the synthesis of ribozymes that specifically target the HIV genome at specific genes. In the catalytic cleavage of the HIV1 genome, synthesized hammerhead ribozymes were used, where they were designed primarily to cleave the RNA molecule in the *tat* at gene (atAT) or common exon for tat and rev (TR) [33, 34]. The anti-HIV-1 ribozymes cloned expressed by the T lymphocytes were cloned with LN retroviral vector plasmids which have shown resistance to HIV-1 replication in one of the studies. By contrast, the cells expressing mutant ribozymes supported HIV-1 replication, demonstrating the cleavage of target RNA by functional ribozymes. These studies show that HIV replication inhuman T-lymphocytes can be inhibited by the retrovirally transduced ribozymes found in long, multifunctional transcripts. The ribozyme and expression strategies specified here should be useful for the HIV-1 AIDS gene therapy by gaining resistance to the replication of HIV-1 on cells derived from transduced hematopoietic stem cells [35]. Ribozymes have many important aspects such as site specific cleavage and functional inactivation of target RNAs, multiple site targeting, possibility of inactivation of cellular coreceptors which make them an attractive therapeutic agent for HIV-1 infection [36, 37].

### Utilization of siRNA and RNA interference

RNA interference is a process, where the inhibition of gene expression or translation is carried out by RNA molecules. siRNAs are the class of double-stranded RNA which is also known as silencing RNA. It operates within the RNA interference pathway. The utilization of RNA interference pathway in targeting HIV-1 infection has been enormously explored by various researchers. According to the experiments conducted on “the SCID Hu mouse thymopoiesis model” [38, 39], anti-HIV molecules, such as RNA decoys, ribozymes, and SiRNA, not only target rev and tat, but also CCR5 and CXCR4 that are involved in viral entry [40]. SiRNA suppresses HIV-1 replication by functioning via endogenous RNA interference pathway and was found to be effective in suppressing HIV infection both in vitro and in vivo. However, this therapy was not successful for HIV cure as the HIV genome undergoes a high rate of mutation, and the escaped viral mutants create a major long-term SiRNA therapy problem. Therefore, when used in conjunction with other therapies, such as ribozymes and RNA decoys, this therapy can be much more efficient. Although the combination constructs have shown its efficacy in vitro and in vivo, the effectiveness of this therapy on humans is yet to be explored. SiRNA is also involved in inhibition of viral production by targeting mRNA for the HIV-1 cellular receptor CD4, the viral structural Gag or the green fluorescent protein substituted for the Nef regulatory protein [40, 41].

### Digoxin

**Fig. 3:**
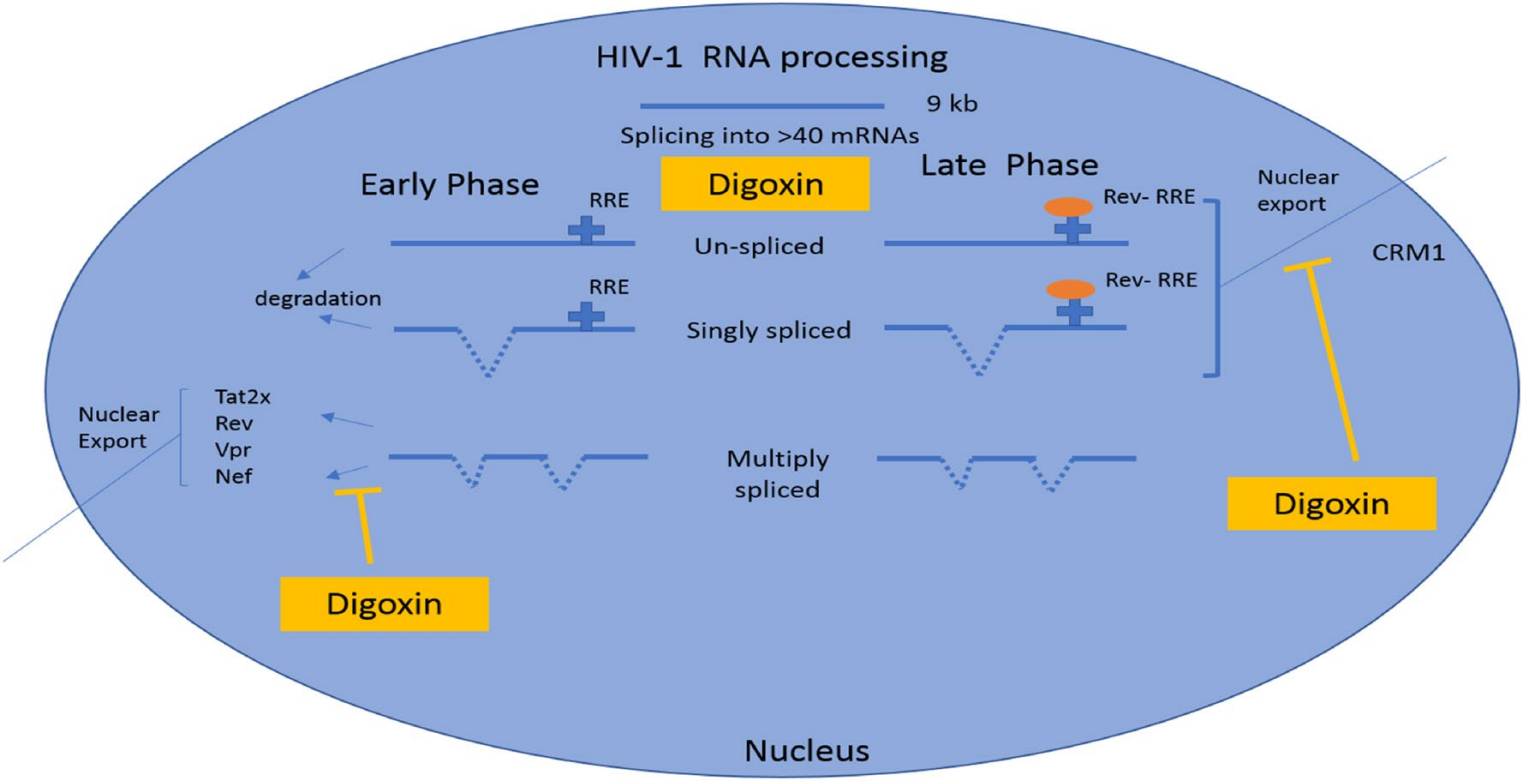
Mechanism of action of digoxin against HIV-1 [42]

Despite its wide usage in cardiac patients suffering from congestive heart failure and other cardiac anomalies, it is found that digoxin shows efficacy against HIV-1 infection [42]. Digoxin hinders the replication of HIV virus by modulating the pre-mRNA splicing events. Some of the mechanisms by which digoxin show its beneficial effect against HIV-1 are, (i) carrying out the over splicing of HIV-1 pre-mRNA which results in the decrease in the levels of both un-spliced and singly spliced mRNA [42] and (ii) digoxin obstructs the nuclear export of RNAs by altering the pre-mRNA splice sites within multiple spliced mRNA (as the binding of mRNA and regulatory proteins such as Rev is hindered). The reduction of incompletely spliced viral mRNA by both mechanisms interrupts the synthesis of various HIV-1 regulatory, accessory as well as structural proteins essential for the formation of new virion assembly. In addition to these mechanisms, digoxin also selectively suppresses the expression of Rev Protein by causing alteration in the splicing events [42]. Although digoxin has been found to be effective in HIV-1 treatment, its usage is questionable due to its narrow therapeutic dose range. In addition to these, as HIV-1 patients are weak and immunocompromised, use of digoxin may subject the patients to more risk than benefits. The below diagram explains the mechanism of action of digoxin in HIV1 treatment (Fig. 3).

### Other strategies trending in the treatment of HIV-1 infection targeting Rev protein

HIV proteins are widely explored for their activities in HIV-1 replication. Out of all the proteins, Rev and Tat are being extensively studied. Although research is being carried out with various strategies (Table 1), and on a positive note, some of the strategies are found to be effective in vitro and in vivo, their effectiveness in humans still needs to be confirmed. The below table (Table 1) explains the various strategies being explored against HIV-1 infection by targeting Rev protein.

**Table 1:** Various strategies involved in targeting Rev protein in HIV-1 infection

Strategy	Mechanism of action	Reference
Romidepsin	It is a cyclic tetrapeptide HDAC inhibitor which is an investigational drug in Phase-2 clinical trials for HIV-1 infection treatment in combination with ART. It acts as a latency-reversing agent	[43]
ADAR-1 protein	ADAR1 mediated inhibition of viral protein synthesis occurs at a post-transcriptional step of viral replication, at the step of nuclear export of viral Gag, Pol and Env mRNA. This effect of ADAR1 on nuclear export was seen to be due to ADAR1-induced A-to-G mutations on Rev and RRE region on env. These ADAR1 induced A-to-G mutations on HIV-1 rev and env mRNA correlated with inhibition of virus replication, production and infectivity	[44]
Pyronin Y	Pyronin Y is an intercalating dye which has an ability to inhibit the complex formation between the HIV-1 Rev protein and RRE-containing RNA	[45]
Aminoglycoside antibiotics	Certain aminoglycoside antibiotics, in particular neomycin B, can block binding of the HIV Rev protein to its viral RNA recognition element and found to be effective in vitro and in vivo	[46]
miRNA	Human miR-186, 210, and 222 directly regulate the human genes Dicer1, HRB, and HIV-EP2, thus downregulating HIV-1 replication and miRNA biogenesis	[47]
Benfluron	Benfluron inhibits RRE-Rev ribonucleoprotein formation by binding to the RRE RNA, and blocks Rev action and HIV-1 transcription	[48]
Leptomycin B	It inhibits the nucleo-cytoplasmic translocation of Rev at nanomolar concentrations. Rev dependent export of mRNA into the cytoplasm is also blocked by leptomycin B, which inhibits Rev-dependent, but not Rev-independent gene expression in a short-term transfection assay	[49]
Anti-Rev antibodies	An anti-Rev single-chain variable fragment (SFv) moiety can be efficiently expressed, using murine retroviral vectors, in human T lymphocytic cell lines as well as in primary human blood mononuclear cells (PBMC). Both mixed cellular populations and cell clones, transduced with the anti-Rev SFv, demonstrated significant resistance to productive human immunodeficiency virus type 1 (HFV-l) replication	[50]
Rev aptamers	The lead aptamer designated as A-1 was fused to a siRNA that targeted the HIV-1 tat/rev RNAs that encode early regulatory proteins required for replication. The resulting chimeric construct (Ch A-1) is designed to deliver the siRNA to HIV-1-infected cells, resulting in targeted, RNAi-mediated knockdown of Tat/Rev expression	[35]
Small molecule inhibitors (791, 833, 891)	All three compounds resulted in significant reduction in the accumulation of both Tat and Rev thus inhibit HIV-1 protein expression in vitro by blocking expression of both early (Rev, Tat) and late (Gag, Env) HIV-1 proteins	[42]

## Conclusion

As the existing therapies for HIV-1 infection fail to treat the persistent HIV-1 infections and associated rebound viremia, there is a great necessity for approaches that target HIV virus at its initial stages of life cycle. Targeting regulatory proteins such as Rev, Tat is one such approach which is capable of combating HIV-1 infections both in early and late phases of HIV life cycle. Multiple strategies, such as gene therapies, immunotherapy, and vaccines, are being screened recently for targeting latent HIV infections, but none of them have been capable of completely eradicating HIV. However, the progress in research and development in combating HIV-1 infection is commendable and gives us a hope that HIV-1 infections may vanish from the world in the coming future.

## Abbreviations

ADAR1: Adenosine deaminase acting on RNA
ART: Anti-retroviral therapy
ARV: Anti-retro viral drugs
CCR5: Chemokine receptor 5
CRISPR: Clustered regulatory interspaced short palindromic repeats
CXCR4: C–X–C chemokine receptor type 4
FDA: Food Drug Administration
FDC: Fixed dose combinations
HAART: Highly active anti retroviral therapy
HIV-1: Human immunodeficiency virus 1
HIV-EP2: Human immunodeficiency virus enhancer binding protein 2
HRB: HIV1 Rev binding protein
InIs: Integrase inhibitors
LTR: Long-terminal repeat
MHC 1: Major histocompatibility complex 1
Nef: Negative regulating factor
NHEJ: Non-homologous end joining
NNRTISs: Non-nucleoside reverse transcriptase inhibitors
NRTIs: Nucleoside reverse transcriptase inhibitors
PBMC: Peripheral blood mononuclear cells
PIs: Protease inhibitors
PIC: Pre-integration complex
RRE RNA: Rev response element RNA
SiRNA: Small interfering RNA
